# Development of Eco-Sustainable PBAT-Based Blown Films and Performance Analysis for Food Packaging Applications

**DOI:** 10.3390/ma13235395

**Published:** 2020-11-27

**Authors:** Arianna Pietrosanto, Paola Scarfato, Luciano Di Maio, Loredana Incarnato

**Affiliations:** Department of Industrial Engineering, University of Salerno, Via Giovanni Paolo II, 132, 84084 Fisciano (SA), Italy; pscarfato@unisa.it (P.S.); ldimaio@unisa.it (L.D.M.); lincarnato@unisa.it (L.I.)

**Keywords:** biodegradable polymers, PBAT/PLA, blown films, food packaging, toughness

## Abstract

In this work, eco-sustainable blown films with improved performance, suitable for flexible packaging applications requiring high ductility, were developed and characterized. Films were made by blending two bioplastics with complementary properties—the ductile and flexible poly(butylene-adipate-*co*-terephthalate) (PBAT) and the rigid and brittle poly(lactic acid) (PLA)—at a 60/40 mass ratio. With the aim of improving the blends’ performance, the effects of two types of PLA, differing for viscosity and stereoregularity, and the addition of a commercial polymer chain extender (Joncryl^®^), were analyzed. The use of the PLA with a viscosity ratio closer to PBAT and lower stereoregularity led to a finer morphology and better interfacial adhesion between the phases, and the addition of the chain extender further reduced the size of the dispersed phase domains, with beneficial effects on the mechanical response of the produced films. The best system composition, made by the blend of PBAT, amorphous PLA, and the compatibilizer, proved to have improved mechanical properties, with a good balance between stiffness and ductility and also good transparency and sealability, which are desirable features for flexible packaging applications.

## 1. Introduction

Currently, the packaging sector is among the major consumers of plastic materials and, in this field, conventional non-biodegradable polymers are widely employed for their desirable properties. However, they become a major source of waste after use due to their poor biodegradability. Therefore, the use of biodegradable polymers for packaging applications represent an effective strategy to decrease the quantity of plastics waste sent to landfill and facilitate bio-waste collection and organic recycling, therefore reducing the plastics disposal problems [1,2,3].

Poly(butylene adipate-*co*-terephthalate) (PBAT) is a biodegradable random copolymer, consisting of aromatic and aliphatic chains. Among biodegradable polymers, it stands out for its very high ductility and flexibility, which make PBAT particularly interesting for packaging applications such as plastic bags and wraps. However, its poor stiffness, low transparency, and low seal strength until now have limited its use [4]. In this context, the melt blending of PBAT with another bioplastic could represent an effective and economic way to improve its properties without compromising its biodegradability. Poly(lactic acid) (PLA), is a bio-based and biodegradable polyester with good processability and interesting properties in the packaging field that could also be customized by varying the relative content of the D and L isomers [5,6]. It has high transparency and complementary mechanical properties to PBAT, exhibiting high stiffness but also high brittleness [7,8]. Therefore, the melt blending of these two polymers, by varying the mass ratio of PLA and PBAT in the blend, can be a useful strategy to modulate and tailor the performance of the final product from a rigid and brittle material (100% PLA) to a ductile and flexible one (100% PBAT) [9]. Several authors have explored this possibility, mainly with the aim to reduce the brittleness of blends having PLA as a matrix phase, obtaining interesting advantages in terms of the mechanical performances of such blends. Commercial examples of PLA- and PBAT-based blends are already available on the market under the trade name of Ecovio; however, their high price limits the diffusion of these materials on a large-scale [10]. Furthermore, since the exact composition of these blends is confidential, the study of the factors affecting PLA/PBAT blends properties can be of great importance in order to further enhance their performance, therefore improving their diffusion in the large-scale market. In this context, since PLA and PBAT are not thermodynamically miscible, although they have very close solubility parameters, researchers have also evidenced the necessity to control the morphology and the interfacial adhesion between the phases in order to gain optimized properties of the final product [11].

Among the different strategies, the incorporation of multifunctional chain extenders containing epoxy groups proved to be an effective way to improve the compatibility between these polymers [12,13,14,15,16]. Joncryl^®^ is a commercial food-grade multifunctional epoxy chain extender, specifically designed for biodegradable polymers and PET. Its compatibilization and chain extension mechanisms are obtained by the formation of in situ block copolymers, through the reaction of the epoxy groups with the terminal groups of polyesters, specifically by epoxy ring-opening and subsequent hydrogen abstraction from hydroxyl and carboxylic acid groups [17,18]. The average number of epoxy groups per chains (functionality), which usually ranges between 4 and 9, influence the final branching degree of the formed copolymer and therefore, the processability of the final system, as previously reported [18].

Several studies, performed on PBAT/PLA blends with PLA as a matrix phase, demonstrated that the incorporation of this chain extender, at concentrations ranging from 0.25 to 1 wt %, leading to the formation of extended and branched chains and, at the same time, to the formation of a PLA–Joncryl–PBAT copolymer, which is placed at the interface between the two phases, thus enhancing the interfacial adhesion [19,20] and the final performances of the resulting blends [20,21,22,23]. However, few works deal with the effect of Joncryl^®^ in blends with PBAT as a matrix phase and the results reported in the literature have not shown a relevant improvement of the mechanical properties of the resulting systems [24,25,26,27].

Moreover, other strategies could also be adopted in order to improve the performance of immiscible blends. In fact, it is widely known that, among several factors, the stereoregularity of a component polymer and the relative viscosity of the two phases can considerably affect the compatibility and the properties of blend systems, as demonstrated for several conventional and biodegradable blend systems [28,29,30]. As regards PLA and PBAT blends, the effect of PLA tacticity on the blend performance has not been considered until now, and the effect of the viscosity ratio was evaluated only by Lu et al. [31]. They observed that in blends made by dispersed PBAT in the PLA matrix (PBAT/PLA 30/70 *w*/*w*), an increase in the dispersed phase/matrix viscosity ratio led to an increase in size of PBAT domains and to a decrease in the interfacial tension between the two polymers. However, also in this case, there are no studies on the effect of the viscosity ratio for blends of PLA and PBAT in which PBAT is the matrix phase.

In this work, we focused our attention on PBAT/PLA blends with a high content of PBAT: these systems could be of great importance for applications where high ductility is required, such as packaging applications at low storage temperature, as demonstrated in our previous work [9]. In particular, here, we intend to investigate the effects of both PLA stereoregularity and PLA/PBAT relative viscosity on the morphology and properties of blend systems, in which PBAT is the matrix phase. To this aim, two commercial types of PLA, with different viscosities and stereoregularity, have been used as blend constituents. Moreover, the effect of the addition of Joncryl^®^ chain extender on the developed morphologies and properties of the produced films was also considered.

## 2. Materials and Methods

### 2.1. Materials

PLA 4032D (semicrystalline, D-isomer content = 1.5 wt %, Mw ~241,700 g/mol, specific gravity = 1.24 g/cm^3^, *T*_m_ = 155–170 °C), named as PLA1, and PLA 4060D (amorphous, D-isomer content = 12 wt %, Mw ~190,000 g/mol, specific gravity = 1.24 g/cm^3^), named as PLA2, were supplied by NatureWorks LLC (Minnetonka, MN USA). Ecoworld PBAT 009 (density = 1.26 g/cm^3^, *T*_m_ = 110–120 °C), composed of 29 wt % of adipic acid, 26 wt % of terephthalic acid, and 45 wt % of 1,4-butanediol, was manufactured by Jin Hui Zhaolong (Lüliang, China). A multifunctional epoxy chain extender named as Joncryl ADR-4368C (referred to as Joncryl in the following), with Mw = 6800 g/mol, epoxy equivalent weight = 285 g/mol, and functionality >4, was supplied by BASF (Ludwigshafen, Germany). All the materials comply with USA FDA and EU regulations for food contact.

### 2.2. Preparation of the Films

PLA1, PLA2, and PBAT pellets were dried under vacuum at 70 °C for 16 h prior to processing. The polymers and the chain extender were mixed at the compositions reported in Table 1. Each mixture was melt blended in a Collin ZK25 co-rotating twin-screw extruder (COLLIN Lab & Pilot Solutions GmbH, Maitenbeth, Germany, *D* = 25 mm, *L/D* = 42) at a constant speed of 100 rpm (mass flow equal to 51–53 g/min) and with a temperature profile ranging from 140 to 180 °C from the hopper to the die. Then, the neat polymers and the two blends were dried under vacuum at 70 °C for 16 h before processing. In order to ensure a homogeneous distribution of the material in the extruder head, the blown films were prepared using two extruders GIMAC (Caserta, Italy, *D* = 12 mm, *L/D* = 24) of a multilayer co-extrusion blown film plant. The processing temperature at different zones was set from 190 to 135 °C, the screw speed was 25 rpm (mass flow equal to 17–18 g/min), and the take-up speed was 3 m/min. Films were produced with a blow-up ratio (BUR) and a take-up ratio (TUR) equal to 1.7 and 20, respectively, and an average thickness of 23 ± 0.8 µm.

### 2.3. Films Characterization

The rheological properties in oscillatory mode of the extruded pellets of the neat materials and the blends were measured using an ARES rotational rheometer (Rheometrics, Inc., Piscataway, NJ, USA). Samples were dried under vacuum at 70 °C for 16 h prior to testing. Tests were performed with a parallel-plate geometry (*d* = 25 mm) with a gap of 1mm at 180 °C under a nitrogen atmosphere. A strain sweep test was initially conducted to guarantee the linear viscoelastic regime for each formulation. Thus, all the frequency sweep tests were performed with a strain equal to 5% and with a frequency ranging from 0.1 to 100 rad/s.

The morphology of the blends was analyzed using a field emission scanning electron microscope (FESEM) (LEO 1525 model, Carl Zeiss SMT AG, Oberkochen, Germany). First, film samples were cryo-fractured and then, coated with a thin gold layer (Agar Auto Sputter Coater mod. 108 A, Stansted, UK) at 30 mA for 160 s to improve their conductivity. After, their cross sections parallel to the transversal direction (TD) were scanned by FESEM.

Thermal analysis was carried out using a Differential Scanning Calorimeter (DSC mod. 822, Mettler Toledo, Columbus, OH, USA) under a nitrogen gas flow (100 mL/min). Three scans were performed; samples were heated from −70 to 200 °C with a speed of 10 °C/min and held at 200 °C for 5 min. After, they were cooled at −70 at 10 °C/min and heated again to 200 °C at 10 °C/min. The crystallinity degree of PLA, *X*c, was calculated as follows:

*X*_c_ = (Δ*H*_m_ − Δ*H*_cc_)/(Δ*H*m^0^ × φ_i_) × 100 (1)

where Δ*H*_m_ and Δ*H*_cc_ (J/g) are the heat of melting and heat of cold crystallization, respectively, Δ*H*m_0_ is equal to 93.6 J/g [9] for PLA, and φ_i_ is the relative weight fraction of PLA in the blend.

Tensile testing of the blown films was performed on a SANS dynamometer equipped with a 100 N load cell. The rectangular shape specimens (width = 12.7 mm and length = 30 mm) were extended at a crosshead speed set according to ASTM D822 standard [32]. The mechanical properties were evaluated in the machine direction (MD). All the data are the average of at least ten measurements.

The transparency of the films was evaluated according to ASTM D1746-03 [33]. Films were cut into rectangular shapes and placed on the internal side of a spectrophotometer cell. Then, the transmittance was measured using a UV–vis spectrophotometer (Lambda 800, PerkinElmer, Waltham, MA, USA) at 560 nm. Three replicates of each film were tested. The percent transparency (*TR*) was calculated as follows:

*TR* = *T*_r_/*T*_0_ × 100 (2)

where *T*_r_ is the transmittance with the specimen in the beam and *T*_0_ is the transmittance with no specimen in the beam.

Hot-tack strength was evaluated using a heat-sealing machine (mod. HSG-C by Brugger, Munich, Germany) equipped with a Hot Tack Device. A pair of film ribbons with 15 mm width were fitted between two heated bars and hot-pressed together at 80 N for 0.5 s, at 85 °C, according to ASTM F1921 [34]. The hot-tack data were measured just after the films were heat-sealed and, while still hot, they were pulled apart, recording the weight required to separate the two sealed surfaces. The result is an average of three specimens.

## 3. Results and Discussion

### 3.1. Rheological Analysis

**Figure 1:**
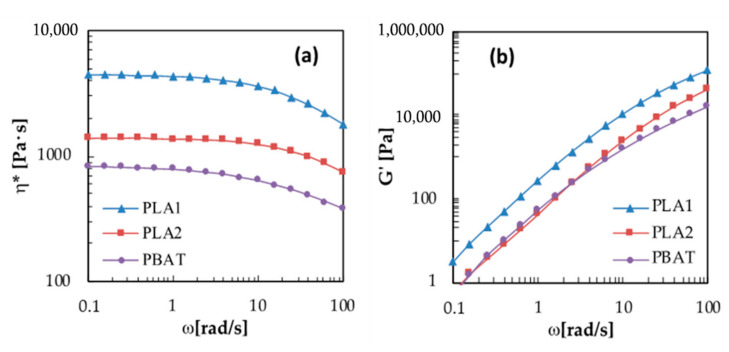
(**a**) Complex viscosity and (**b**) Storage Modulus of the neat polymers.

PBAT, PLA1, and PLA2 resins were submitted for rheological measurements in order to investigate the differences in their flow behavior. The complex viscosity (η*) and storage modulus (*G’*) curves, obtained at 180 °C, are compared in Figure 1a,b. The graphs of Figure 1 show that all the neat polymers exhibit at low frequency a Newtonian plateau and a shear thinning behavior at higher ω values. Both types of PLA have complex viscosity markedly higher than that of PBAT in the whole analyzed frequency range. Moreover, PLA1 has higher viscosity and storage modulus than PLA2, as expected. In fact, PLA1 has lower D-lactide content (higher stereoregularity), which increases the secondary forces in the polymer melt, and higher molecular weight [35,36]. Computing the viscosity ratios (i.e., viscosity of dispersed phase / viscosity of matrix phase) of the two pairs of polymer melts at different frequencies, we obtained η*_PLA1_/η*_PBAT_ from 5.4 (ω = 0.1 rad/s) to 4.8 (ω = 100 rad/s) and η*_PLA2_/η*_PBAT_ from 1.7 (ω = 0.1 rad/s) to 2.0 (ω = 100 rad/s). Differences in the viscosity values can affect the morphology of the resulting blend system [29,30,31]. If the viscosity ratio of the melts is much higher than one, a coarse morphology of the blend should be expected, while if it is close to one, it is possible to achieve a finer morphology. Therefore, in this case, a better mixing can be supposed for the blends with PLA2 than those with PLA1.

**Figure 2:**
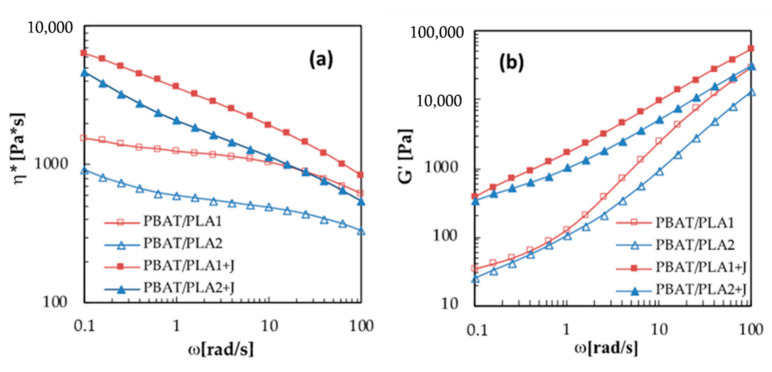
(**a**) Complex viscosity and (**b**) storage modulus of the blends.

The complex viscosity and storage modulus of the blends are reported in Figure 2a,b. Both the uncompatibilized blends showed, at 0.1 rad/s, a complex viscosity between those of the respective neat materials. Particularly, the PBAT/PLA2 blend had a lower complex viscosity with respect to PBAT/PLA1 in all the analyzed frequency range, according to the different viscosities of the neat PLA resins. Moreover, the first system also exhibited a more accentuated shear thinning behavior compared to the second one and to the respective neat materials, which could be attributed to the occurrence of reactions (e.g., transesterification) between PLA2 and PBAT, which resulted in a wider molecular weight distribution [37,38].

For both the blends, the addition of the chain extender resulted in an increase in the complex viscosity values at all frequencies and in a strong enhancement of the shear thinning behavior, as predictable from the literature [19,20,21]. It is widely known that zero-shear viscosity is related to the molecular weight of the polymer and the enhanced shear thinning behavior is attributable to the increase in chain branching and molecular weight distribution. Therefore, our rheological analysis suggests that in the used processing conditions, the addition of the multifunctional epoxy compatibilizer led to the formation of the PLA–Joncryl–PBAT copolymer, having an increased length and a more branched structure compared to the PBAT and PLA in the uncompatibilized blends, as found also by others in reactive extrusion experiments of PLA and PBAT with an epoxy compatibilizer [13,18,20].

As reported in Figure 2b, the storage modulus of PBAT/PLA1 blend was also higher with respect to PBAT/PLA2, according to the storage modulus of the respective neat materials. However, the PBAT/PLA1 blend exhibited a shoulder of *G’* at low frequency values, which is due to the additional elastic response generated by the surface tension of the dispersed domains in the continuous matrix [9], which suggests higher surface tension between PBAT and PLA1 with respect to PLA2.

The addition of the compatibilizer led to an increase in the storage moduli for both the systems, which was more relevant for low frequency values, as a result of the longer relaxation times of the compatibilized blends, which are owed by the formation of longer and more branched chains and to a more entangled structure [19].

Moreover, the incorporation of Joncryl led to a reduction in the shoulder at low frequency observed for the blend containing PLA1, therefore suggesting that the presence of the chain extender led to a reduction in the surface tension between PLA1 and PBAT [15].

**Figure 3:**
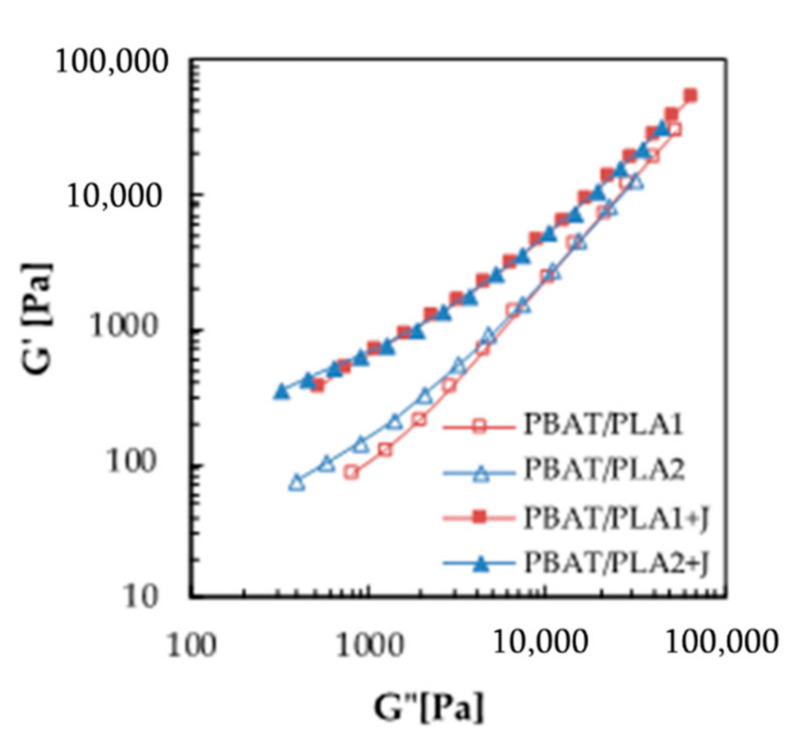
Plot of *G’* versus *G”* for the blended systems.

In Figure 3, the storage modulus is plotted versus the dissipative one for all the blended systems.

It has been demonstrated that the plot of *G’* versus *G”* can be used as a criterion of compatibility of a blended system, since it gives composition-independent correlations for compatible blends and composition-dependent correlations for incompatible blends [39]. According to this affirmation, as reported in Figure 3, both the compatibilized systems showed the same correlation between *G’* and *G’’*, while the *G’–G”* correlations were not coincident for the un-compatibilized blends, which is a further confirmation of the compatibilization effect of Joncryl. Moreover, the PBAT/PLA2 system exhibited a *G’–G”* correlation closer to the compatibilized blend with respect to the PBAT/PLA1 one, suggesting, together with the complex viscosity and storage modulus curves, a higher compatibility between PLA2 and PBAT compared to PLA1.

### 3.2. Morphology

**Figure 4:**
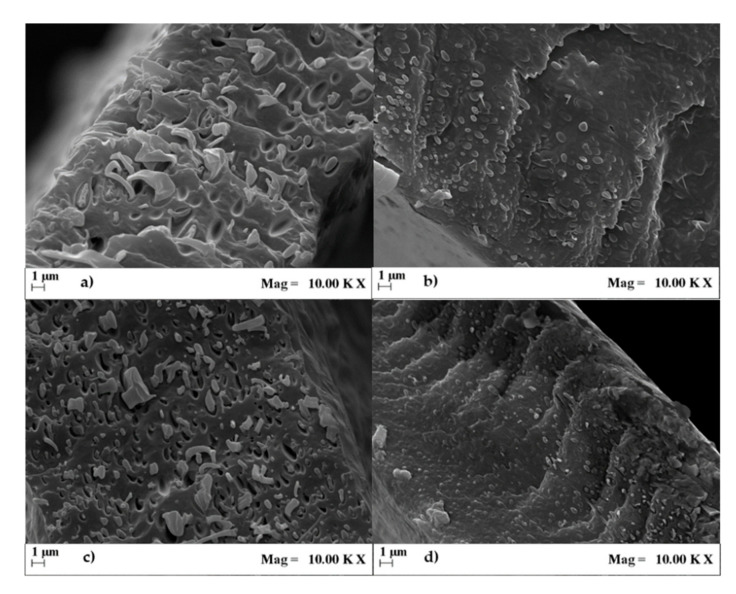
SEM picture of the fracture surfaces of (**a**) PBAT/PLA1, (**b**) PBAT/PLA2, (**c**) PBAT/PLA1 + J, and (**d**) PBAT/PLA2 + J.

The effects of both the type of PLA and the incorporation of Joncryl compatibilizer on the PBAT/PLA blend morphology were investigated by means of FESEM analysis. The images taken on cryo-fractured film sections are reported in Figure 4. Both the uncompatibilized blends (Figure 4a,b) showed the typical two-phase morphology of immiscible systems, with PLA domains dispersed in the PBAT matrix with quite uniform distribution and average size. However, in the PBAT/PLA1 blend, the droplets had bigger dimensions (>2 µm) than in the PBAT/PLA2 one (<1 µm) and were pulled out by cryo-fracturing, leaving empty cavities in the PBAT matrix. These findings are coherent with the rheological results. In fact, since PLA2 has a complex viscosity closer to PBAT compared to PLA1, it was possible to have, in the same process conditions, a better mixing for the blend containing PLA2 that was traduced in a finer and more homogeneous morphology, with fewer and smaller voids and a more ambiguous interface compared to the PBAT/PLA1 blend, indicative of a better interfacial adhesion. Two factors can contribute to these findings. One is the formation of PLA–PBAT mixed chains (copolymers) that can be generated during the melt blending of PBAT and PLA: the reaction is influenced by the melt viscosity ratio of the neat polymers and by their molecular mobility in the melt state, as demonstrated by other authors [37]. The other is the lower stereoregularity of PLA2 compared to PLA1. Because of this, PLA2 has a more flexible polymer chain; therefore, it can be hypothesized that the higher segmental mobility may enhance the polar group accessibility, consequently increasing the possibility of hydrogen bonding to PBAT. Additional analyses are necessary to clarify this point, which will be investigated in further work.

Both the compatibilized blends (Figure 4c,d) kept the two-phase morphology; however, the addition of Joncryl led to a relevant reduction in the dispersed phase size without changing its shape, compared to the respective uncompatibilized blend. This indicated a decrease in the interfacial tension and a higher compatibility between the two constituents due to the in situ formation of the PLA–Joncryl–PBAT copolymer based on the combination of PLA and PBAT chains, which is placed at the interface between the two phases, as demonstrated by others on blends of PLA and PBAT of different compositions [16,18,24]. Moreover, the effect of the chain extender seemed more evident for blends containing PLA1 than for those containing PLA2, likely owing to the weaker interactions between PLA1 and PBAT that could be enhanced by the addition of the compatibilizer.

### 3.3. Thermal Properties

**Figure 5:**
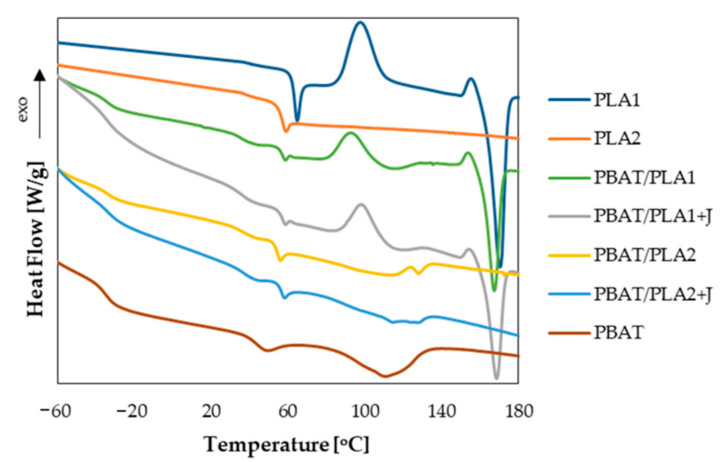
Thermograms of the film related to the first heating scan.

The thermal properties of a polymeric system considerably influence the performance of the final product. Therefore, the thermal properties of the films were also investigated. The thermograms and the main thermal parameters of the films related to the first heating scan are reported in Figure 5 and Table 2, respectively.

From the thermograms, it is evident that PLA1 is a semi-crystalline polymer, while PLA2 is completely amorphous; differences in their thermal behavior owe to their different content of D-isomer (see Materials section). They both exhibited a glass transition temperature around 60 °C and PLA1 also showed a cold crystallization and a melting peak at 97 and 170 °C, respectively. A small exothermic peak just before the melting point can be observed in the thermogram of PLA1, which is related to the transition from the α’ crystal form to the more stable α ones [40]. Neat PBAT exhibited a lower glass transition temperature compared to PLA, and two melting points—the first one related to the melting of the butylene adipate fraction (around 49 °C) and the second one to the co-crystallization of butylene adipate units into butyl terephthalate crystals (around 111 °C) [41].

All the blends showed both the glass transitions of the neat polymers, representative of their immiscibility [9,42], and all the main thermal transitions of the respective components. As regards the un-compatibilized blends, the blending with PBAT led to a reduction in the cold crystallization temperature of PLA1 and an increase in its crystallinity degree, since PBAT increases the crystallization rate of PLA, as previously reported [25]. However, the melting peak of PBAT in the PBAT/PLA1 film was partially hidden by the cold crystallization of PLA; therefore, the calculation of its crystallinity degree was not possible.

The blends containing PLA2 exhibited a double PBAT endothermic peak, which can be attributable to the formation of two separate crystalline phases resembling those of the homopolymers: polybutylene adipate and poly butyl terephthalate [41]. Since this isodimorphic behavior of PBAT in PBAT/PLA2 blends is different from the neat PBAT and in the blends containing PLA1, which exhibited one melting peak at 111–115 °C, it can be supposed that PLA2 has influenced the crystallization behavior of PBAT, as a confirmation of the highest interaction between PBAT and PLA2.

The presence of Joncryl led to an increase in the cold crystallization temperature of PLA1 for the PBAT/PLA1 + J film and to a slight decrease in the melting enthalpies of PBAT for both the compatibilized blends, which is attributable to the increase in the molecular weight and branching density of the polymers that hindered the crystallization process [22,25]. Moreover, the presence of the compatibilizer also increased the glass transition of PLA2, a sign of the reduced mobility of the amorphous chain segments, which have a more rigid structure [43].

### 3.4. Mechanical Properties

**Figure 6:**
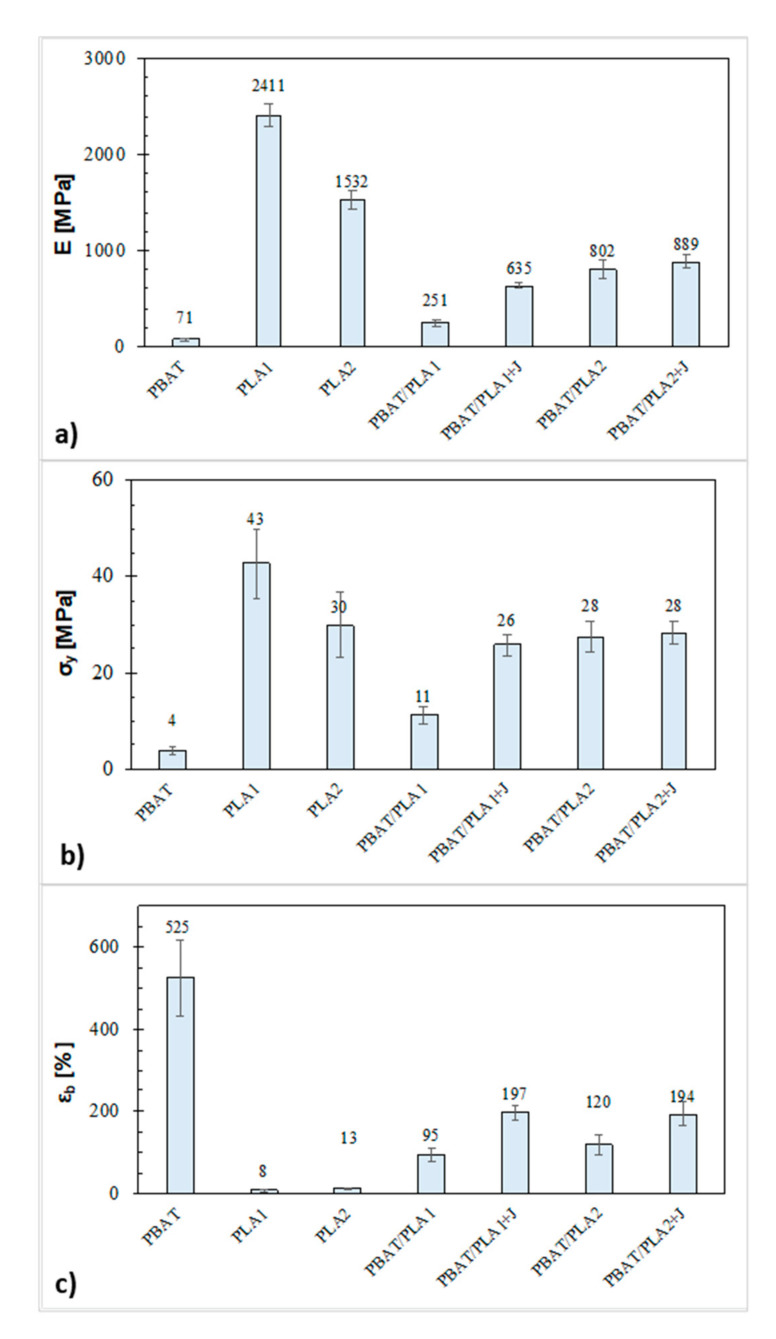
(**a**) Elastic modulus, (**b**) yield stress, and (**c**) elongation at break of the films.

The results obtained from the tensile tests are reported in Figure 6. It is evident that PLA and PBAT have complementary mechanical properties: the first is rigid and brittle, while the latter is flexible and tough [9]. Moreover, comparing the two types of PLA, it can be observed that PLA1 has a higher elastic modulus and yield stress than PLA2 too, which is related to the amorphous nature of PLA2 due to its high D-isomer content that led to a worsening of the materials’ rigidity [44].

All the blends showed mechanical properties between those of the corresponding neat materials. From the comparison of two uncompatibilized systems, it is evident that the PBAT/PLA2 blend, although based on the less rigid PLA, had a considerably higher elastic modulus (800 MPa) and yield stress (28 MPa) than the PBAT/PLA1 one, which exhibited an elastic modulus and yield stress of 251 and 11MPa, respectively. Since both the elastic modulus and the yield stress are greatly affected by the nature of the interface of a multi-phase system and the blend morphology [45], this result is a further confirmation of a better compatibility and higher strength of the interactions between PBAT and PLA2 with respect to PLA1, as observed in the rheological and morphological analysis. These findings point out that the viscosity ratio and the nature of the interface between the blend constituents had a more important role than the individual resin properties in determining the blend strength and stiffness. Nevertheless, as regards the ductility of the films, the use of different PLA did not considerably affect the elongation at break.

With the addition of Joncryl, the elastic modulus and yield stress of the system containing PLA1 were more than tripled and doubled, respectively, since the addition of the chain extender enhanced the strength of the interactions between PLA1 and PBAT and therefore, considerably improved the interface of the resulting system, while, for the system containing PLA2, the effect of the compatibilizer was not so effective. In particular, the elastic modulus slightly increased, and the yield stress did not change, because the PBAT/PLA2 blend already showed a good phase adhesion, also without the addition of the compatibilizer, as revealed by the morphological analysis. However, although greatly improved, the stiffness and the strength of the PBAT/PLA1 + J blend (*E* = 635 MPa and σ_y_ = 26 MPa) were still lower than those of PBAT/PLA2 + J (*E* = 889 MPa and σ_y_ = 28 MPa).

Moreover, the addition of Joncryl also led to an improvement in the ductility of the systems, whose value was doubled for both the blends, owing to the reduction in the dispersed phase domains, as found in the SEM analysis.

Up to now, similar benefits due to Joncryl addition were reported only for PLA/PBAT with PLA as a matrix phase [20,21,22,25,26], whereas investigations on these blends with PLA as a dispersed phase showed a worsening of the mechanical performance. Only Nunes et al. [24] reported that the addition of 0.5 wt % Joncryl led to an improvement of the ductility of the systems, but with a negative impact on the materials’ rigidity, since the concentration used was not enough to increase the interfacial adhesion between the phases. Therefore, from the comparison of the present results with the literature data, it turns out that the relative viscosity between the phases, the content of the compatibilizer, and the blending process conditions play a key role in determining the effectiveness of the chain extension reaction, the morphology, and thus, the final performance of the resulting system.

On the whole, among all the blends investigated in this study, the system that allowed the achievement of the best mechanical properties was PBAT/PLA2 + J, which exhibited a good compromise between stiffness and ductility.

### 3.5. Optical Properties

Transparency is an important physical property of packaging films, and transparent film materials are highly desirable for a number of packaging applications [46]. The transparency values are displayed in Table 3. Both the types of PLA films exhibited high transmittance; in particular, PLA2 was slightly more transparent than PLA1 due to its amorphous nature. On the other hand, light transmission of the PBAT film was almost prevented, in accordance with the literature data [47]. The blends showed transparency values between those of the neat materials. PBAT/PLA1 films had lower transparency than PBAT/PLA2 and the difference in the transparency values of the blends was markedly higher than the neat PLAs. This is attributable to the fact that, as reported in the thermal analysis, blending semi-crystalline PLA with PBAT, led to an increase in its crystallinity degree, therefore resulting in a lower transparency of the PBAT/PLA1 films with respect to PBAT/PLA2.

Moreover, the addition of the compatibilizer slightly increased the transparency of both the systems due to the reduction in the crystallinity degree of the polymers.

The transparency values of the PBAT/PLA2 films, with and without the compatibilizer, were comparable with those reported for commonly used non-biodegradable plastics such as polyethylene (PE) [48]; therefore, even if they are lower than those of PLA, they can be considered as acceptable for packaging application requiring see-through properties.

### 3.6. Hot-Tack Measurements

Sealability is one of the key performance requirements for flexible packaging that allows packages to be made at high packaging speeds and keeps the product secure [49].

As reported in Table 4, amorphous PLA exhibited the highest seal strength, while the semi-crystalline one proved to be not sealable. In fact, it is widely known that the sealability of a material is also linked to its crystallinity degree and amorphous polymers have higher chain mobility on the surface of the film, leading to higher diffusion and thus, higher adhesion strength [50].

Consequently, since the seal strength of PBAT was markedly lower than PLA2, blends containing PLA1 had a seal strength lower than 100 g/15 mm, while PBAT/PLA2 blends proved to have a seal strength close to PLA2, which was not considerably influenced by the incorporation of the compatibilizer. Few works have reported on the sealability of bioplastics, particularly, as regards PBAT/PLA blends, only Tabasi et al. [51] investigated the hot-tack behavior of PBAT/PLA blends with PLA as a matrix phase, obtaining higher values of the hot-tack strength due to the higher content of PLA in the blend. However, the hot-tack strength of PBAT/PLA2 blends was comparable with those reported for PE films [52]; therefore, PBAT/PLA2 films proved to also have adequate seal properties as flexible packaging materials.

## 4. Conclusions

In this work, eco-sustainable PBAT/PLA blown packaging films, having high toughness and being suitable for direct food contact, were successfully produced.

In order to optimize film performances, PBAT/PLA blends in a 60/40 mass ratio were produced starting from two commercial types of PLA with different viscosities and stereoregularity, with and without the use of the multifunctional epoxy chain extender Joncryl, so as to investigate the effects of the different viscosity ratio and stereoregularity of the polymer melts and the chain extension reaction on the blend constituent compatibility and on the final film properties.

Rheological and morphological investigations have shown that the PBAT/PLA2 blend, whose PLA had a lower stereoregularity and a viscosity closer to PBAT than the PLA1 blend, has a finer dispersion and distribution of the dispersed PLA phase and a stronger interfacial adhesion between phases. Consequently, the PBAT/PLA2 system shows better mechanical performance than the corresponding blend with PLA1, especially in terms of stiffness, even if based on the less rigid PLA2.

Moreover, the use of an amorphous PLA, such as PLA2, allowed the obtaining of better transparency and hot-tack strength, which are other key properties of flexible packaging films.

For both the investigated blend systems, but to a greater extent for the PBAT/PLA1 blend, the addition of Joncryl promoted interactions between the two constituents. This resulted in a finer morphology and better mechanical response of the compatibilized systems, in terms of elastic modulus, yield stress, and elongation at break. However, only minor changes were measured in terms of transparency and hot-tack strength.

On the whole, the best performing system was the PBAT/PLA2 + J film, which exhibited the best compromise in terms of stiffness and ductility, with mechanical performances (*E* = 889 MPa, σ_y_ = 28 MPa, ɛ_b_ = 194%) and transparency and hot-tack strength (43% and 600 g/15mm, respectively), comparable with those reported for commonly used non-biodegradable plastics.

## Figures and Tables

**Table 1:** Blends compositions.

Sample	PBAT Content (phr)	PLA 4032 Content (phr)	PLA 4060 Content (phr)	Joncryl Content (phr)
PBAT	100	-	-	-
PLA1	-	100	-	-
PLA2	-	-	100	-
PBAT/PLA1	60	40	-	-
PBAT/PLA1 + J	60	40	-	1
PBAT/PLA2	60	-	40	-
PBAT/PLA2 + J	60	-	40	1

**Table 2:** The main thermal properties of the films related to the first heating scan: Glass Transition Temperature (*T*g), Cold Crystallization Temperature (*T*_cc_), Cold Crystallization Enthalpy (Δ*H*_cc_), Melting Temperature (*T*_m_), Melting Enthalpy (Δ*H*_m_), and Crystallinity degree (*X*_c_).

Sample	*T*g ^PBAT^ (°C)	*T*g ^PLA^ (°C)	*T*_cc_ (°C)	Δ*H*_cc_ (J/g)	*T*_m1_^PBAT^ (°C)	*T*_m2_^PBAT^ (°C)	Δ*H*_m_^PBAT^ (J/g)	*T*_m_^PLA^ (°C)	Δ*H*_m_^PLA^ (J/g)	*X*_c_^PLA^ (%)
PBAT	−35.4	-	-	-	48.8	110.6	17.2	-	-	-
PLA1	-	63.4	97.5	28.5	-	-	-	170.1	30.1	1.7
PLA2	-	56.3	-	-	-	-	-	-	-	-
PBAT/PLA1	−34.1	57.1	92.6	8.1	41.1	115.4	2.3	167.1	13.8	15.2
PBAT/PLA1 + J	−33.9	57.2	98.1	8.9	41.0	116.5	1.7	168.2	14.1	13.9
PBAT/PLA2	−33.3	54.6	-	-	42.3	111.8–124.1	7.2	-	-	-
PBAT/PLA2 + J	−34.2	56.3	-	-	42.5	114.4–125.3	5.3	-	-	-

**Table 3:** Transparency of the films.

Sample	Transparency (%)
PBAT	5.8 ± 0.7
PLA1	89.7 ± 0.2
PLA2	91.0 ± 0.8
PBAT/PLA1	8.2 ± 0.1
PBAT/PLA1 + J	12.4 ± 0.1
PBAT/PLA2	40.7 ± 0.7
PBAT/PLA2 + J	43.0 ± 0.9

**Table 4:** Hot-tack measurements of the films.

Sample	Hot-Tack Strength (g/15 mm)
PBAT	125 ± 5
PLA1	-
PLA2	650 ± 10
PBAT/PLA1	-
PBAT/PLA1 + J	-
PBAT/PLA2	610 ± 10
PBAT/PLA2 + J	600 ± 15
